# Viral diversity and blood-feeding patterns of Afrotropical *Culicoides* biting midges (Diptera: Ceratopogonidae)

**DOI:** 10.3389/fmicb.2023.1325473

**Published:** 2024-01-05

**Authors:** Edwin O. Ogola, Armanda D. S. Bastos, Inga Slothouwer, Caroline Getugi, Josephine Osalla, Dorcus C. A. Omoga, Dickens O. Ondifu, Rosemary Sang, Baldwyn Torto, Sandra Junglen, David P. Tchouassi

**Affiliations:** ^1^International Centre of Insect Physiology and Ecology (icipe), Nairobi, Kenya; ^2^Department of Zoology and Entomology, University of Pretoria, Pretoria, South Africa; ^3^Institute of Virology, Charité Universitätsmedizin Berlin, Corporate Member of Free University Berlin, Humboldt-University Berlin, and Berlin Institute of Health, Berlin, Germany

**Keywords:** arbovirus surveillance, vertebrate hosts, next generation sequencing, *Culicoides* biting midges, *Goukovirus*, *Pacuvirus*, *Orthobunyavirus*, Iflavirus

## Abstract

**Introduction:**

*Culicoides* biting midges (Diptera: Ceratopogonidae) are vectors of arboviral pathogens that primarily affect livestock represented by Schmallenberg virus (SBV), epizootic hemorrhagic disease virus (EHDV) and bluetongue virus (BTV). In Kenya, studies examining the bionomic features of *Culicoides* including species diversity, blood-feeding habits, and association with viruses are limited.

**Methods:**

Adult *Culicoides* were surveyed using CDC light traps in two semi-arid ecologies, Baringo and Kajiado counties, in Kenya. Blood-fed specimens were analysed through polymerase chain reaction (PCR) and sequencing of cytochrome oxidase subunit 1 (*cox1*) barcoding region. *Culicoides* pools were screened for virus infection by generic RT-PCR and next-generation sequencing (NGS).

**Results:**

Analysis of blood-fed specimens confirmed that midges had fed on cattle, goats, sheep, zebra, and birds. *Cox1* barcoding of the sampled specimens revealed the presence of known vectors of BTV and epizootic hemorrhagic disease virus (EHDV) including species in the Imicola group (*Culicoides imicola*) and Schultzei group (*C. enderleni*, *C. kingi*, and *C. chultzei*). *Culicoides leucostictus* and a cryptic species distantly related to the Imicola group were also identified. Screening of generated pools (11,006 individuals assigned to 333 pools) by generic RT-PCR revealed presence of seven phylogenetically distinct viruses grouping in the genera *Goukovirus*, *Pacuvirus* and *Orthobunyavirus*. The viruses showed an overall minimum infection rate (MIR) of 7.0% (66/333, 95% confidence interval (CI) 5.5-8.9). In addition, full coding sequences of two new iflaviruses, tentatively named Oloisinyai_1 and Oloisinyai_2, were generated by next-generation sequencing (NGS) from individual homogenate of *Culicoides* pool.

**Conclusion:**

The results indicate a high genetic diversity of viruses in Kenyan biting midges. Further insights into host-vector-virus interactions as well as investigations on the potential clinical significance of the detected viruses are warranted.

## Introduction

*Culicoides* or biting midges (Diptera: Ceratopogonidae) are tiny insects measuring about 1–3 mm in length, commonly identified by unique wing pigmentation and macrotrichia pattern (Mathieu et al., 2012; Kirk-Spriggs and Sinclair, 2017; Borkent and Dominiak, 2020). They have a global distribution with species important for veterinary and public health mainly grouped into the genus *Culicoides* Latreille (Diptera: Ceratopogonidae) comprising about 1,340 species. The Afrotropical region is home to diverse *Culicoides* species, with over 120 species described in southern Africa alone (Garros et al., 2019; Borkent and Dominiak, 2020). Examples of medically important *Culicoides* species include invasive species of the *Culicoides imicola* complex (*C. imicola, C. brevitarsis and C. bolitinos*) and Schultzei group (*C. enderleni, C. kingi*, and *C. schultzei*) (Bakhoum et al., 2013; Leta et al., 2019).

Both sexes of *Culicoides* feed on plants as a primary energy source, but females also blood-feed on vertebrates such as mammals, birds and lizards to acquire additional protein to fertilize their eggs (Martínez-de la Puente et al., 2015; Borkent and Dominiak, 2020). Some *Culicoides* species rely on insect haemolymph as a protein source (Lassen et al., 2012). Further, there is a record of *C. anophelis* and *C. nubeculosus* feeding on engorged mosquitoes (Kremer et al., 1974; Ma et al., 2013). Like other insects, *Culicoides* harbour insect-specific viruses of the genera *Iflavirus* (Langat et al., 2021). Iflaviruses are arthropod-infecting viruses which are not known to be pathogenic to animals and have been largely identified in arthropods of the orders Lepidoptera and Hemiptera (Carrillo-Tripp et al., 2014).

The hematophagous feeding tendency ranks *Culicoides* among arthropods of veterinary and public health importance as they transmit parasites, protozoa and arboviruses that can cause severe disease, such as Schmallenberg virus (SBV) (family *Peribunyaviridae*, genus *orthobunyavirus*) and Bluetongue virus (BTV) (family *Reoviridae,* genus *orbivirus*) (Elbers et al., 2008; Santiago-Alarcon et al., 2012; Elbers et al., 2013; Sick et al., 2019; Žiegytė et al., 2021). Schmallenberg virus is grouped into the simbu serogroup together with Akabane virus (AKV) and Shuni virus (SHUV) and predominantly infects domestic ruminants causing fever, diarrhoea, and serious fetal malformation in gestating cattle and sheep (Saeed et al., 2001). Other viruses in the simbu serogroup include Oropouche virus (OROV) which has been associated with febrile illness in humans and is common in the neotropics (Romero-Alvarez and Escobar, 2018; Gaillet et al., 2021). On the other hand, viruses of the genus *Orbivirus* such as epizootic hemorrhagic disease virus (EHDV) and BTV are known to cause haemorrhagic disease and high morbidity in livestock leading to movement bans and strict trade restrictions which cause huge economic losses (Elbers et al., 2008; Rivera et al., 2021). Outbreaks of *Culicoides*-borne viruses have been common in Europe, for instance SBV in Germany, Italy, Netherlands, UK and Belgium (Hoffmann et al., 2012,; Elbers et al., 2013; Stokes et al., 2018; Ferrara et al., 2023a,b), but there are also reports of EHDV in Canada and North-eastern United States (Stallknecht et al., 2015; Allen et al., 2019). In Africa, BTV is endemic in South Africa, Morocco and Algeria (Youssef et al., 2015; Grewar, 2016; Moustafa et al., 2016; Durr et al., 2017).

The epizootics of *Culicoides*-borne viruses demonstrate the important role of neglected insect groups in veterinary health as outbreaks often mirror distribution of vectors (Purse et al., 2004). While the development and host biting rate of vectors such as *C. enderleni* and *C. imicola* are associated with warm temperatures, colder temperatures distinctly enable sustained EHDV and BTV transmission in an episystem (Grimaud et al., 2019; Mayo et al., 2020; Ferrara et al., 2023a,b). Another factor facilitating sustained virus transmission is the availability of vertebrate hosts that support large insect populations and may also serve as reservoir and maintenance hosts (Kuno et al., 2017). To understand the transmission dynamics of *Culicoides*-borne viruses, it is important to identify blood-meal sources of the vectors and abundant vector species. However, apart from reports highlighting active circulation of BTV serotypes in Kenya, studies on *Culicoides*-borne viruses are generally scarce in Africa (Toye et al., 2013; Onyango et al., 2015; Langat et al., 2021). In this study, we sought to identify *Culicoides*-borne viruses in two arid ecosystems, Baringo and Kajiado counties in Kenya using generic RT-PCR assays and next-generation sequencing (NGS). We further examined the vertebrate blood-meal sources of *Culicoides* in this area and identified abundant species by molecular barcoding.

## Methods

### Study location

**Figure 1:**
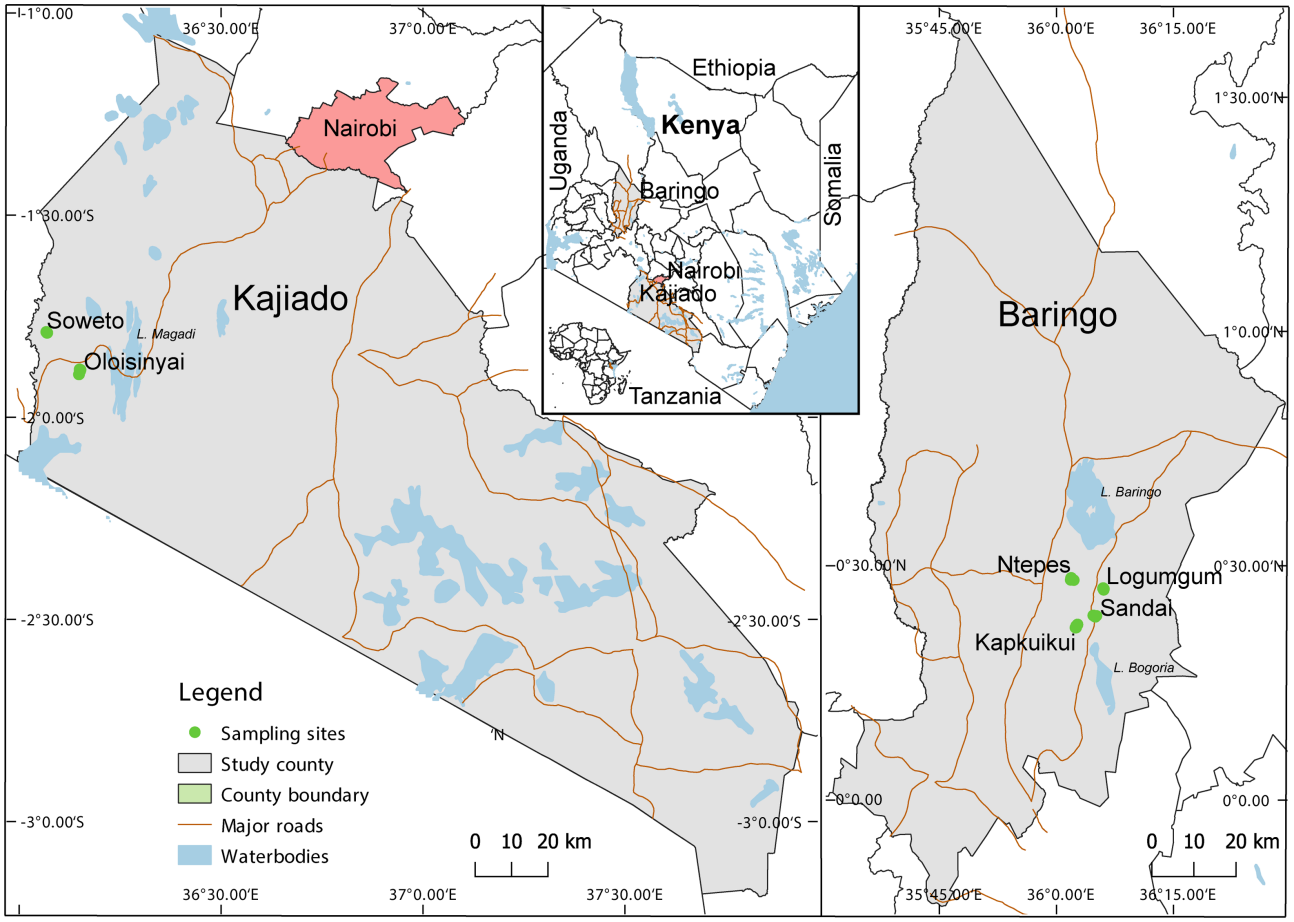
Map showing specimen collection sites in two semi-arid counties Kajiado and Baringo, in Kenya during entomological surveillance activities conducted between August 2019 and July 2020. The map was generated in QGIS 2.12 with shape files provided by Natural Earth (http://www.naturalearthdata.com/) and Africa Open data (https://africaopendata.org/dataset/kenya-counties-shapefile) (QGIS Development Team, 2019).

Adult *Culicoides* were collected between August 2019 and July 2020 in Baringo and Kajiado counties at the end of rainy season and coinciding with peak abundance (Diarra et al., 2014). The study spanned 4 sites, Ntepes, Sandai, Logumgum, and Kapkuikui, in Baringo County, and 2 sites in Kajiado county, namely Soweto and Oloisinyai (Figure 1). The 2 study areas have similar semi-arid ecologies. Baringo had mean daily temperatures of 21.4°C, mean daily rainfall of 3.0 mm and a mean relative humidity of 66.8% during the sample collection periods. In Kajiado, the mean daily temperature was 23.7°C, mean daily rainfall was 0.3 mm and mean relative humidity 66.0% during sampling period. Using GIS-coordinates, the weather variable data were retrived from https://power.larc.nasa.gov/, and mean daily temperature and humidity in Kajiado documented using Thermochron iButton (Sunnyvale, CA) attached to each trap. The sparse human population in both areas rely on nomadic pastoralism as the major economic activity. A typical family has large herds of cattle, goat and/or sheep which they move from one place to another in search of water and pasture. The areas also have conservancies haboring wild animals including Kiborgoch community conservancy in Baringo and Olkirimatian conservancy in Kajiado. Some of the common wild animals in the conservancies include zebra, impala antelope and diverse bird species, including ostriches.

### *Culicoides* collection and identification

Centers for Disease Control (CDC) light traps model 512 (John W. Hock Company, Gainesville, United States) were used to catch adult *Culicoides* as part of a larger arbovirus surveillance project. Eight CDC light traps were baited with yeast-produced CO_2_ (Laguna-Aguilar et al., 2012), and deployed overnight from 18:00 h to 06:00 h for 3 consecutive nights at each sampling site. The traps were deployed outdoors with an inter-trap distance of about 25 m geo-referenced positions near farms/animal shelters (in the vicinity of human-settlement) as well as inside wild animal conservancies. After retrieval, the samples were initially taken to a temporary field laboratory. Biting midges were anesthetized with triethylamine and cryopreserved in liquid nitrogen for transportation to the laboratory at *icipe* in Nairobi and stored at −80°C until further analysis. In the laboratory, the *Culicoides* were sorted from other insects on a pre-chilled ice pack under a dissecting microscope (Stemi 2000-C microscope, Zeiss, Germany) (Glick, 1990; Bakhoum et al., 2018). Unfed *Culicoides* were pooled in groups of 2 to 50 according to collection date and sampling site. Engorged (blood-fed) specimen were analyzed individually.

### Homogenization of midge samples

The collected *Culicoides* were homogenized for 30 s in 1.5 mL microcentrifuge tubes containing 2.0 mm zirconia beads and DPBS (Dulbeccos phosphate-buffered saline, pH 7.4) using a Mini-Beadbeater-16 (Biospec, Bartlesville, OK, United States). For each pool, 1 mL of DPBS was used, whereas for individual engorged specimens the volume was reduced to 500 μL. The homogenate was centrifuged for 10 min in a bench top centrifuge (Eppendorf, United States) at 2500 revolutions per minute (rpm) at 4°C. After phase separation, the supernatant was used for virus screening and isolation. The pellet of individual engorged samples were preserved for DNA analyzes inclusive of species identification and blood-meal source determination.

### Molecular identification of engorged *Culicoides* species and blood-meal source detection

DNA was extracted from the homogenate pellets of individual engorged *Culicoides* using DNeasy Blood and Tissue Kit (Qiagen, Hilden, Germany) following manufacturer’s recommendations and stored at −20°C until further use. *Culicoides* species molecular identification involved polymerase chain reaction (PCR) and Sanger sequencing of a 710 bp region of the cytochrome oxidase subunit 1 (*cox1*) gene as previously described (Folmer et al., 1994). To identify different blood meal host sources a different *cox1* fragment was targeted using vertebrate specific primers (Reeves et al., 2018). ExoSAP-IT (USB Corporation, Cleveland, OH, United States) was used to remove unincorporated dNTPs and PCR primers from the amplicons before submitting purified amplicons for Sanger sequencing (Microsynth Seqlab GmbH, Göttingen, Germany). Sequences were edited in Geneious prime and used to query Barcode of Life (BOLD) and GenBank databases (Altschul et al., 1990; Ratnasingham and Hebert, 2007).

### Detection and characterization of viruses

Viral RNA was extracted from 140 μL of the homogenate supernatants using the Viral RNA Mini Kit (Qiagen, Hilden, Germany) as described by the manufacturer. Double-stranded cDNA was synthesized using random hexamer primers (Endoh et al., 2005) and High Capacity cDNA Reverse Transcription (RT) kit (Life Technologies, CA, United States) following the manufacturers’ protocol. Pan-PCR assays targeting the RNA-dependent RNA polymerase (RdRp) gene of peribunyaviruses and phenuiviruses were utilized to screen for viral infections as described earlier (Hermanns et al., 2023). The resulting PCR products were examined on 1.5% agarose gels stained with ethidium bromide and amplicons of the correct size were purified for Sanger sequencing (Macrogen, Amsterdam, Netherlands) using ExoSAP-IT (USB Corporation, Cleveland, OH, United States).

### Virus isolation

Virus-positive samples were inoculated onto semi-confluent monolayers of mammalian Vero E6 (*Ceropithecus aethiops*) and insect C6/36 (*Aedes albopictus*) and KC (*Drosophila melanogaster*) cell lines, as described previously (Junglen et al., 2009). Briefly, Vero E6 cells were maintained in Dulbecco’s modified Eagle’s medium (DMEM)supplemented with 5% fetal calf serum (FCS) and 1% l-glutamine. C6/36 and KC cells were maintained in Leibovitz’s L-15 medium (L-15) supplemented with 5% FCS. Vero E6 cells were maintained in a 5% CO_2_ incubator at 37°C while C6/36 and KC cells were maintained at 28°C incubator without CO_2_. The cells were observed regularly for up to 7 days for occurrence of cytopathic effects (CPE). Samples were passaged on fresh cells 8 days after inoculation and a 75 μL aliqout of cell culture supernatant was taken from each sample. The blind passage was repeated 3 times and aliquots from cell culture supernatants from each passage were tested for viral replication.

### Library preparation and next-generation sequencing

Sequencing libraries were constructed using the KAPA HyperPlus kit (Roche Diagnostics, Rotkreuz, Switzerland) following the manufacturers’ instructions. Sequencing was performed on the Illumina MiSeq platform (Illumina, United States) as described (Marklewitz et al., 2019). Low-quality reads and adaptor sequences were removed from paired-end reads using BBDuk (filterk = 27, trimk = 30; http://jgi.doe.gov/data-and-tools/bb-tools/). After quality control, the cleaned reads were *de novo* assembled using Spades v3.11 implemented in Geneious Prime (Kearse et al., 2012). The resulting contigs were queried against the NCBI reference sequence database using BLASTn search.

### Sequence and phylogenetic analyzes

Sequences were analyzed using Geneious prime (Kearse et al., 2012). Obtained sequences were compared to publicly available sequences in BOLD and GenBank (Altschul et al., 1990; Ratnasingham and Hebert, 2007). Sequences were aligned to related sequences using MAFFT as implemented in Geneious prime (Kearse et al., 2012). Maximum likelihood (ML) phylogenetic analyzes were executed using PhyML v. 2.2.4 in Geneious prime with each of the aligned *cox1* nucleotide datasets. Nodal support was assessed by 1,000 bootstrap replicates using standard parameters (Guindon et al., 2010). Flavivirus and iflavirus genome organization were predicted using interProScan executed in Geneious prime (Kearse et al., 2012; Jones et al., 2014).

### Statistical analysis

Metadata including *Culicoides* trap catches, sampling area, GPS coordinates, collection date, trapping method and abdominal status were captured in Microsoft Excel (2020) and relative abundance of *Culicoides* in sampling areas analyzed in R version 4.1.2 using funrar package (R Core Team, 2016). Minimum infection rate (MIR) was determined using PooledInfRate (https://github.com/CDCgov/PooledInfRate) under the assumption that there was at least one infected specimen in every positive *Culicoides* pool (Ségard et al., 2018). The relative abundance of *Culicoides* was estimated binomially and evaluated by Chi square tests at 95% confidence intervals in R version 4.1.2 (R Core Team, 2016).

## Results

### *Culicoides* abundance in Kenya

A total of 11,006 *Culicoides* were collected from the six sampling sites in the two counties using CDC light traps (Table 1). More *Culicoides* were collected from Baringo County (55.9%, *n* = 6,149) than from Kajiado County (44.1%, *n* = 4,857) (Table 1). Overall, the majority of *Culicoides* specimens were collected in Oloisinyai, Kajiado County (42.0%; *n* = 4,622). In Baringo County, Logumgum (23.8%, *n* = 2,620) and Kaptombes (13.3%, *n* = 1,462) yielded the highest numbers of *Culicoides.* Seventy-four individuals (0.7% of the total collection) were engorged (blood-fed) specimens.

**Table 1:** Relative abundance of *Culicoides* sampled from Baringo and Kajiado counties, Kenya.

County	Habitat type	Sampling site	*n* (%)	Blood-fed (%)
Baringo (55.9%, *n* = 6,149)	Animal conservancy	Kaptombes	1,462 (13.3)	34 (45.9)
	Farm/animal shelter	Logumgum	2,620 (23.8)	17 (23.0)
		Ntepes	891 (8.1)	1 (1.4)
		Kapkuikui	825 (7.5)	16 (21.6)
		Sandai	351 (3.2)	1 (1.4)
		***n* (%)**	**6,149 (55.9)**	**69 (93.2)**
Kajiado (44.1%, n = 4,857)	Animal conservancy	Oloisinyai	4,622 (42.0)	5 (6.8)
	Farm/ animal shelter	Soweto	235 (2.1)	0 (0.0)
		***n* (%)**	**4,857 (44.1)**	**5 (6.8)**
***n* (%)**			**11,006 (100.0)**	**74 (100.1)**

*n*, number of specimens. Bold values highlight the total number of specimen in sub-sections.

### Blood-meal sources of engorged *Culicoides* species

**Figure 2:**
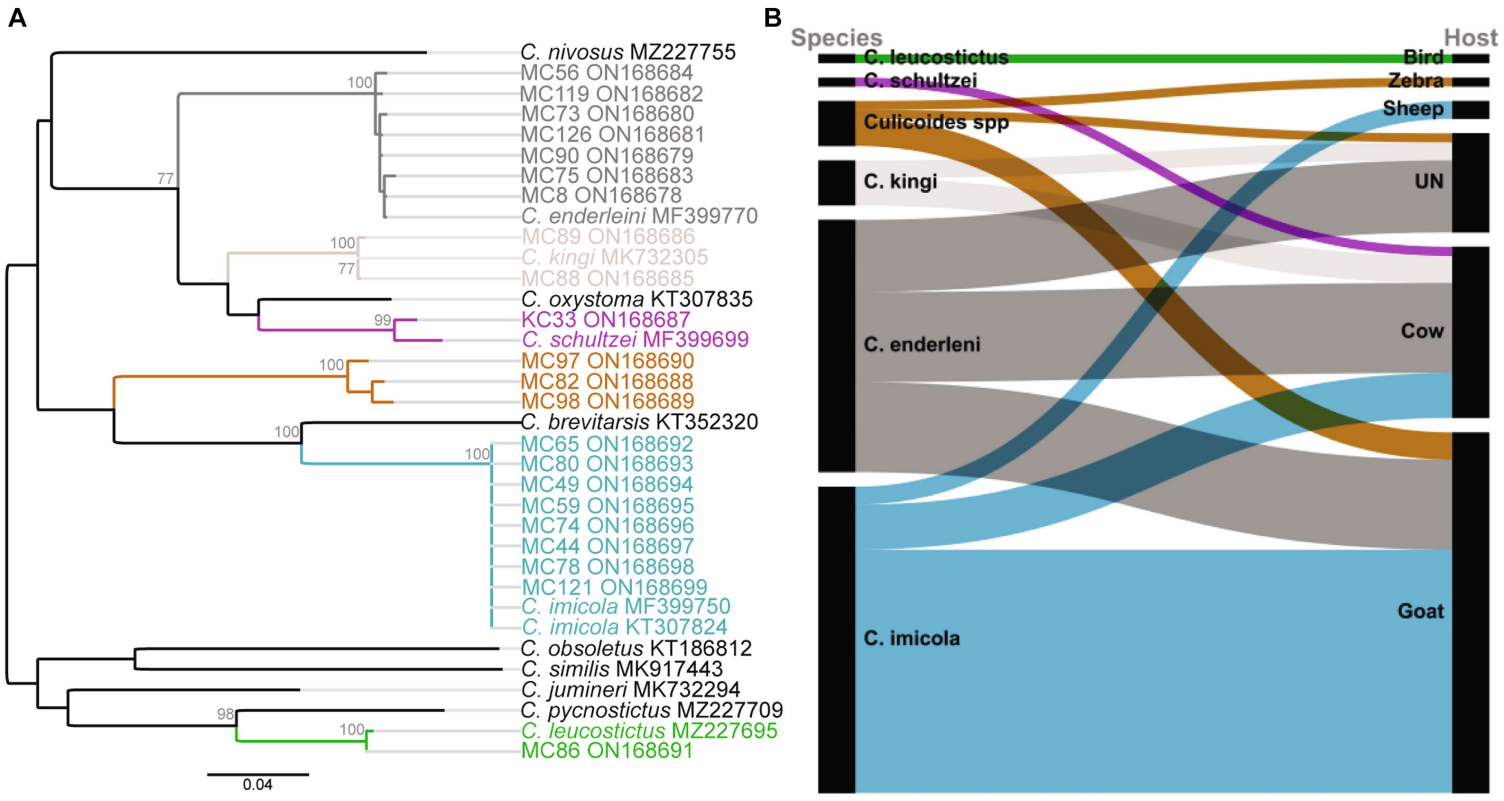
Phylogenetic relationship of identified *Culicoides* species and their blood-feeding patterns. **(A)** Maximum likelihood phylogenetic analysis based on the *Cox1* (nt 710 bp) gene inferred with PhyML v. 2.2.4 using the GTR substitution model. Bootstrap values of 70% and above are shown. **(B)** Alluvial diagram showing *Culicoidies* species blood-meal sources. The diagram was visualized in RAWGraphs (Mauri et al., 2017). UN: Unknown blood-meal source (identification was not successful).

Seventy-four blood-fed specimens mainly originating from Baringo (93.2%, *n* = 69) and only few from Kajiado county (6.8%, *n* = 5) were identified to species by PCR. Most prevalent species were *C. imicola* (45.9%, *n* = 34) followed by *C. enderleni* (37.8%, *n* = 28). Other species identified in the blood-fed cohorts included *C. kingi* (6.8%, n = 5), *C. leucostictus* (1.4%, *n* = 1) and *C. schultzei* (1.4%, *n* = 1). Five samples could not be identified to species level by *cox1* gene analyzes. The sequences of these 5 samples showed maximum nucleotide similarity of 85–86% to *C. brevitarsis* and *C. imicola*, and grouped in a distinct monophyletic clade in distant relationship to the Imicola group (Figure 2A). Among the collected blood-fed specimen, the highest species richness was observed in Kaptombes, Baringo County. Only one species (*C. enderleni*) was found in Sandai and Ntepes in Baringo County. *Culicoides enderleni* was common at all sampling sites, while *C. schultzei* was identified in Oloisinyai, Kajiado County, only.

In total, blood-meal sources were successfully identified from 63 *Culicoides* specimens (85.1%, 63/74; Table 2). Five different vertebrate blood-meal hosts were identified including livestock such as cattle (*Bos taurus*), goat (*Capra hircus*) and sheep (*Ovis aries*), and wild animals exemplified by zebra (*Equus burchelli boehmi*) and birds (*Crinifer piscator*) (Table 2; Figure 2B). *Culicoides imicola* showed the most diverse blood-meal sources having fed on cattle, goat and sheep followed by *C. enderleni* which had fed on cattle and goat. The other species *C. kingi* and *C. schultzei* were found to have fed on cattle only while *C. leucostictus* blood meals were from birds. Analysis of sequence chromatograms did not show evidence for mixed blood-meal sources. No human blood-meal source was identified, despite the trapping conducted in the vicinity of human-settlements.

**Table 2:** Summary of blood-meal sources identified in blood-fed *Culicoides.*

County	Sampling site	Culicoides species	n (%)	Vertebrate host					
				Goats	Cattle	Sheep	Birds	Zebras	UN
Baringo	Kapkuikui	*C. enderleni*	7	4	0	0	0	0	3
		*C. imicola*	7	7	0	0	0	0	0
		*Culicoides* sp.	2	2	0	0	0	0	0
	Kaptombes	*C. enderleni*	6	1	5	0	0	0	0
		*C. imicola*	22	16	4	2	0	0	0
		*C. kingi*	2	0	0	0	0	0	2
		*C. leucostictus*	1	0	0	0	1	0	0
		*Culicoides* sp.	3	1	0	0	0	1	1
	Logumgum	*C. enderleni*	12	5	3	0	0	0	4
		*C. imicola*	5	4	1	0	0	0	0
	Ntepes	*C. enderleni*	1	0	1	0	0	0	0
	Sandai	*C. enderleni*	1	0	0	0	0	0	1
Kajiado	Oloisinyai	*C. enderleni*	1	0	1	0	0	0	0
		*C. kingi*	3	0	3	0	0	0	0
		*C. schultzei*	1	0	1	0	0	0	0
*n* (%)			74	40 (54.1)	19 (25.7)	2 (2.7)	1 (1.4)	1 (1.4)	11 (14.9)

*n*: numbers of blood-fed *Culicoides*; UN: Unknown blood-meal source (identification was not successful).

### Identification of phenuiviruses, peribunyaviruses and iflaviruses

All collected 11,006 *Culicoides* specimens were tested in pools (*n* = 333, ≤50 *Culicoides*/pool) for infections with phenui- and peribunyaviruses. Iflaviruses were detected during analysis of NGS data from *Culicoides* homogenates. An overall minimum infection rate (MIR) of 7.0% (66/333, 95% confidence interval (CI) 5.5–8.9) was found (Table 3). Highest MIR were registered in Sandai (MIR = 47.0, 95% CI 18.3–107.8) and Ntepes (MIR = 11.8, 95% CI 5.4–22.2), both in Baringo County (Table 3).

**Table 3:** Viral detections at the six sampling sites in Baringo and Kajiado counties, Kenya.

County	Sampling	No. of pools	No. of positive	MIR (95% CI)			
	Site	Tested	Pools (%)	All positives	Phenuiviruses	peribunyaviruses	Iflaviruses
Baringo	Kapkuikui	53	4 (1.2)	5.2 (1.6–12.0)	4.0 (1.0–10.3)	0	0
	Kaptombes	75	5 (1.5)	3.6 (1.3–7.8)	2.9 (0.9–6.7)	0	0
	Logumgum	72	7 (2.1)	2.8 (1.2–5.5)	2.8 (1.2–5.5)	0	0
	Ntepes	19	8 (2.4)	11.8 (5.4–22.2)	8.2 (3.2–16.6)	2.4 (0.4–7.3)	0
	Sandai	8	7 (2.1)	47.0 (18.3–107.8)	21.2 (7.5–47.1)	6.7 (1.1–20.7)	0
Kajiado	Oloisinyai	100	34 (10.2)	9.1 (6.4–12.6)	7.5 (5.1–10.6)	0	0.4 (0.1–1.3)
	Soweto	6	1 (0.3)	4.8 (0.3–21.0)	4.8 (0.3–21.0)	0	0
**Total**		**333**	**66 (19.8)**	**7.0 (5.5–8.9)**	**5.7 (4.3–7.3)**	**0.7 (0.3–1.4)**	**0**

MIR, minimum infection rate; CI, confidence interval.

**Figure 3:**
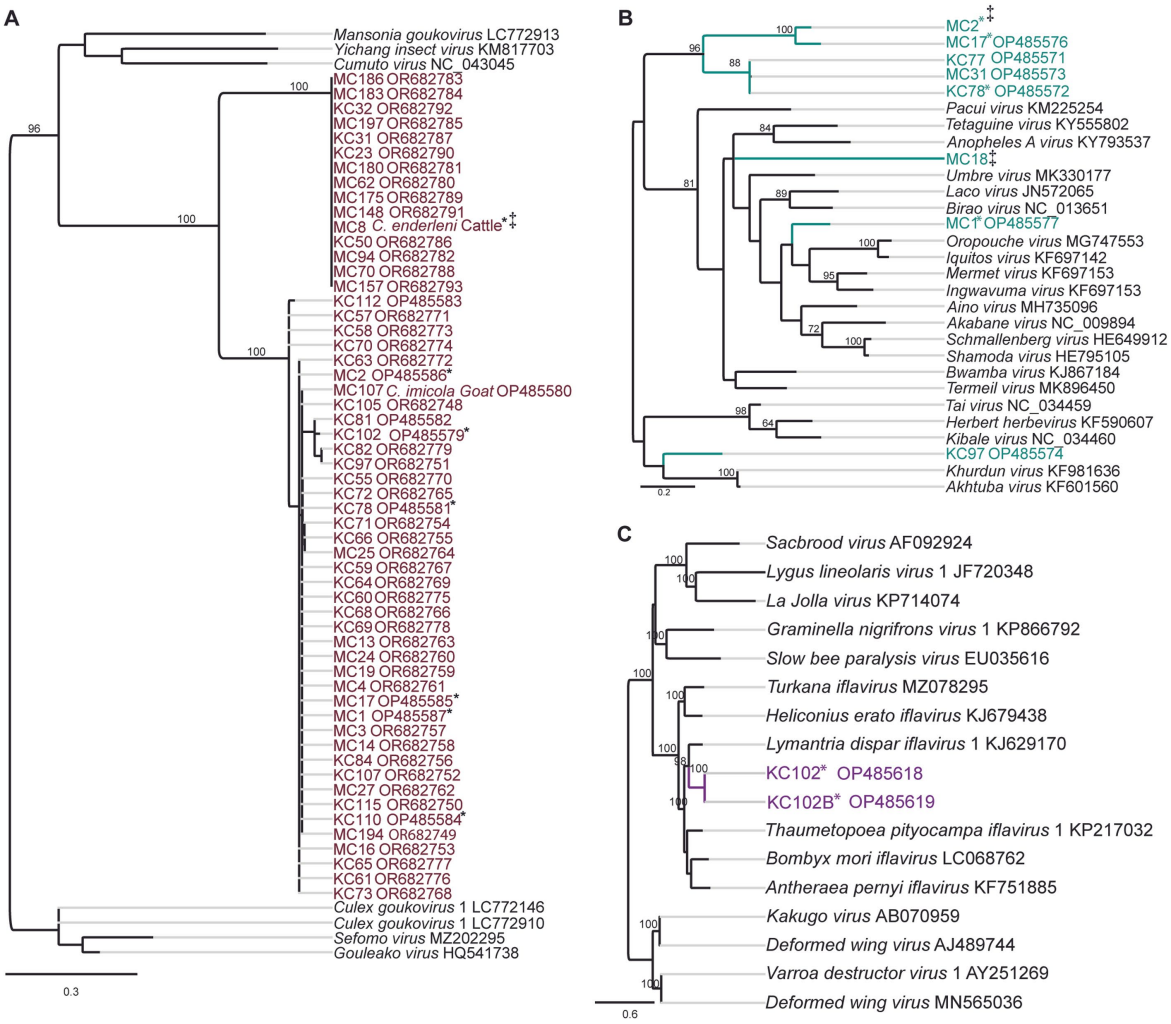
Maximum likelihood RNA-dependent RNA polymerase (RdRp) gene phylogenies depicting the genetic relatedness of **(A)** goukoviruses and samples sequenced in the present study; **(B)** peribunyaviruses and members of the *Peribunyaviridae* family; **(C)** iflaviruses and members of the *Iflaviridae* family. The phylogenetic trees were inferred with PhyML v. 2.2.4. *: Samples with mixed infection; ‡: sequence not deposited to GeneBank due to short length.

Two potential *Goukovirus* species were detected in 56 samples (MIR of 5.7, 95% CI 4.3–7.3, 56/333). The viral sequences were detected at all sampling sites in Baringo and Kajiado counties. Detections were made in *C. enderleni* that had fed on cattle and in *C. imicola* with a blood-meal from a goat (Figure 3A). The 2 potential *Goukovirus* species showed 98–100% and 89–100% nucleotide identities among each at the RdRp gene of 521–233 nucleotides in length. Maximum pairwise amino acid identities of 57 to 71% were found to *Gouleako* virus described in mosquitoes from Cote d’Ivoire (Supplementary Figure S1) (Marklewitz et al., 2011). The phylogenetic tree revealed that the 56 viruses characterized in this study clustered within a monophyletic clade sister to *Gouleako* goukovirus.

Eight RdRp gene sequences that fell within the family *Peribunyaviridae* were identified (Figure 3B; MIR of 0.7%; 95% CI 0.2–1.4, 8/333). BLAST analysis showed that the sequence of the sample MC1 from Sandai, Baringo county, shared 90% amino acid identity to Bahig virus from the Tete serogroup that was isolated from birds in Egypt (Shchetinin et al., 2015). Sample KC97 from Oloisinyai, Kajiado was distantly related to the clade comprising Akhtuba and Khurdun viruses isolated from birds in Russia (Figure 3B) (Al’kovskhovskiĭ et al., 2013). Another sample MC18 was distantly related to Pacui virus (genus *Pacuvirus*) detected in Brazilian rodents (Rodrigues et al., 2014). The remaining samples from Baringo and Kajiado counties clustered together forming a unique monophyletic clade. A distance matrix of pairwise similarity scores of detected viruses and representative peribunyaviruses is shown in Figure S2. There was no peribunyavirus detection in blood-fed specimens.

**Figure 4:**
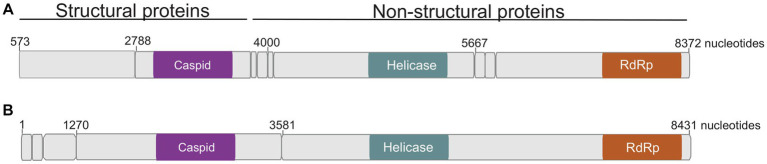
Genome organization of detected Oloisinyai_1 and Oloisinyai_2 iflaviruses. Colored sections highlight regions of functional significance. **(A)** Oloisinyai_1 virus; **(B)** Oloisinyai_2 virus.

Seventeen *Culicoides* homogenate pools positive by Pan-PCR assay were randomly selected and analyzed by NGS. Analysis of NGS data from revealed full coding sequences of two novel iflaviruses (tentatively named Oloisinyai_1 and Oloisinyai_2) in sample KC102 collected from Oloisinyai (Kajiado County) with overall MIR of 0.2% (95% CI 0.0–0.6, 2/17). Comparison of the two sequences against the GenBank database using BLASTx showed 80–82% amino acid similarity to *Lymantria dispar iflavirus 1* isolated in the US (Carrillo-Tripp et al., 2014) (Figure 3C). The detected viral sequences had 85% nucleotide similarity to each other. Genome annotation of Oloisinyai_1 and Oloisinyai_2 revealed a single-stranded positive sense RNA [ssRNA (+)] genome and a genomic organization that includes a capsid protein at N-terminal end and non-structural proteins (helicase and RNA-dependent RNA polymerase) at the C-terminal end (Figure 4). Attempts to isolate the detected phenuiviruses, peribunyaviruses and iflaviruses in KC, C6/36, and Vero E6 cell lines were not successful.

## Discussion

*Culicoides* are vectors of several arboviral pathogens of veterinary and public health importance (Elbers et al., 2008; Romero-Alvarez and Escobar, 2018; Sick et al., 2019; Gaillet et al., 2021). Similar to earlier association of *Culicoides* abundance with warm temperatures, the high *Culicoides* collection in Baringo and Kajiado counties can be attributed to the favorable weather conditions observed in study sites (Grimaud et al., 2019; Mayo et al., 2020). Although the present study involved barcoding of a limited number of blood-fed *Culicoides* (*n* = 74), it revealed the presence of established/known vectors including members of Imicola group (*C. imicola*) and Schultzei group (*C. enderleni, C. kingi*, and *C. schultzei*) (Bakhoum et al., 2013). The rarely encountered species *C. leucostictus* and a cryptic *Culicoides* sp. distantly related to the Imicola group species (*C. imicola* and *C. brevitarsis*) were also identified (Table 2; Figure 2A)*. Culicoides imicola* was the most abundant blood-fed *Culicoides.* This finding is similar to an earlier survey in Senegal showing higher collections of *C. imicola* in traps set near farms/animal shelters suggesting that animal shelters provide dung important for successful breeding of the vector (Acevedo et al., 2010; Diarra et al., 2014, 2015). *Culicoides imicola* is a known vector of BTV and its presence in Kenya provides an important avenue for the virus transmission (Sick et al., 2019). It is noteworthy that there was no record of blood-fed *C. imicola* in Kajiado County. However, the presence of *C. imicola* in the region cannot be excluded since only blood-fed *Culicoides* species were barcoded. *Culicoides* schultzei group species identified in the present study are known vectors of EHDV and BTV (Mellor et al., 1984; Bakhoum et al., 2016) and have been previously described in Kenya (Walker and Boreham, 1976; Cornet and Brunhes, 1994). Their presence highlight the risk of the virus’ transmission and is notable in light of reports of high BTV and EHDV prevalence in western part of the country (Toye et al., 2013). Considering the relatively low numbers of *Culicoides* samples that were barcoded, it is possible that more species and haplotypes could be identified with analysis of larger number of samples. Additional *Culicoides* species characterization would provide valuable information on vector species composition in the region.

*Culicoides* have diverse blood-meal sources. Therefore, information about their blood-feeding pattern is important in elucidating vector-host interactions and *Culicoides*-borne virus eco-epidemiology. Overall, our data confirmed five blood-meal hosts including cattle, goats, sheep, zebras, and birds in agreement with previous studies which reported diverse hosts for *Culicoides* species sampled in Romania (Tomazatos et al., 2020). All *Culicoides* species demonstrated mammophilic blood-feeding habits except *C. leucostictus* which had fed on birds (Table 2; Figure 2B). While ornithophilic tendencies in *C. leucostictus* has been reported before, there is little information on the role of the species in virus transmission (Scheffer et al., 2012). At each sampling site, there appeared to be a correlation between available vertebrate hosts and the blood-meal source detected. For example, at Kaptombes there is a wildlife conservancy inhabited by zebra explaining their detection in *Culicoides* blood-meals. The finding that *Culicoides* were feeding on livestock hosts such as cattle, goat and sheep confirm opportunistic feeding habits, underscoring the role of large animal herds serving as blood-meal sources for vectors (Table 2; Figure 2B). Although hosts availability seemed to influence the observed blood-feeding pattern, it is interesting that human blood-meals were not detected, an indication of potential impact on veterinary rather than human health by *Culicoides*-associated viruses in these ecological settings. A blood-meal source could not be established for 14.9% (*n* = 11) of the blood-fed *Culicoides* (Table 2). The blood-meal analysis involved nested PCR using 3 primer pair combinations of which one, targeting a shorter *cox1* gene of 244 bp, is ideal for detecting degraded host DNA (Reeves et al., 2018). Therefore, blood-meal source identification failure can be attributed to highly degraded host DNA due to advanced digestion in the insect gut. Nevertheless, the study shows that *Culicoides* species in the two counties take diverse blood-meal sources and that the vertebrate hosts can potentially act as reservoirs of *Culicoides*-borne viruses. However, the observed blood-feeding pattern should be interpreted with caution due to the small sample size. A wider study focused on blood-fed *Culicoides* would provide greater insight into *Culicoides* feeding patterns and vector-host interactions.

Virus screening assays revealed the presence of seven potential virus species belonging to the genera *Goukovirus, Pacuvirus, Orthobunyavirus* and *Iflavirus* (Table 3). The family *Phenuiviridae* represents both medically important viruses such as Rift Valley fever virus and the recently discovered sandfly-borne phleboviruses in Baringo County, as well as insect specific viruses like the *gouleako* virus, prototype of the genus *Goukovirus* (Marklewitz et al., 2011; Tchouassi et al., 2019; Marklewitz et al., 2020). Since the discovery of Gouleako virus, several further mosquito-associated goukoviruses have been described (Lefkowitz et al., 2018). However, information associating *Culicoides* with goukoviruses transmission is scarce. The data presented here expand the list of viruses in the genus, describing detection of uncharacterised goukoviruses identified in *Culicoides*. These viruses had a MIR of 5.7% (95% CI 4.3–7.3, 56/333) and were widely distributed, occurring at all sampling sites (Table 3). Further, the viruses were detected in established vectors of EHDV and BTV (*C. enderleni* and *C. imicola*) that had fed on cattle and goat.

One detected vial sequence (MC1) clustered with the pathogenic Simbu group including SBV, AKV, and OROV (Saeed et al., 2001; Romero-Alvarez and Escobar, 2018; Gaillet et al., 2021). Further, sample KC97 was related to Akhtuba and Khurdun viruses isolated from birds in Russia (Al’kovskhovskiĭ et al., 2013; Rodrigues et al., 2014). These virus detections are not surprising as the genus is probably the most medically relevant one within the family *Peribunyaiviridae* and comprises the majority of *Culicoides*-borne viruses (Lefkowitz et al., 2018). Orthobunyaviruses are known to have a wide vertebrate host range inclusive of humans, livestock and birds. In the present study, there was no detection of orthobunyaviruses in *Culicoides* that had fed on livestock and birds, however, this cannot be ruled out due to the small sample size. Unfortunately, attempts to generate more sequence information of the detected peribunyaviruses failed, as well as virus isolation attempts were not successful. Hence, further studies including genome sequencing and analyzes as well as phenotypic studies with virus isolates in cell culture are needed for further classification and assessment.

In the present study, seventeen samples were analyzed by NGS revealing two insect-specific viruses of genus *Iflaviruses* were detected. The low diversity of detected viruses in relation to a similar study could be attributed to the few representative samples that were analyzed by NGS (Langat et al., 2021). The identified iflaviruses showed 80–82% amino acid similarity to *Lymantria dispar iflavirus* 1 (LdIV1) isolated in the US (Carrillo-Tripp et al., 2014), suggesting that the identified iflaviruses represent novel species in the genus as stipulated in the Executive Committee (EC) 51, 2019 report (Lefkowitz et al., 2018). Apart from recent report of iflaviruses in *Culicoides* in Kenya, reports of *Culicoides* iflaviruses infection in the country remains scarce (Langat et al., 2021).

## Conclusion

This study has provided evidence that established *Culicoides* vectors (*C. imicola, C. enderleni, C. kingi*, and *C. schultzei*) of BTV and EHDV were feeding mainly on livestock host in Kenya. Blood-meal analyzes revealed opportunistic feeding of *Culicoides* species on livestock and wildlife and the potential for spillover of viruses of veterinary importance. Expanded studies on vector-host-virus interactions, inclusive of *Culicoides* blood-meal source determination, virus genome characterization and seroprevalence studies are needed to establish the clinical and modulatory impacts of *Culicoides*-borne viruses.

## Data availability statement

The datasets presented in this study can be found in online repositories. The names of the repository/repositories and accession number(s) can be found in the article/supplementary material.

## Ethics statement

The animal study was approved by Kenya Medical Research Institute Scientific and Ethics Review Unit (KEMRI-SERU). The study was conducted in accordance with the local legislation and institutional requirements.

## Author contributions

EO: Conceptualization, Data curation, Formal analysis, Investigation, Methodology, Software, Validation, Visualization, Writing – original draft, Writing – review & editing. AB: Conceptualization, Resources, Supervision, Writing – review & editing. IS: Writing – review & editing, Formal analysis, Investigation. CG: Formal analysis, Investigation, Writing – review & editing. JO: Writing – review & editing, Formal analysis, Investigation. DOm: Formal analysis, Investigation, Writing – review & editing. DOn: Formal analysis, Investigation, Writing – review & editing. RS: Conceptualization, Funding acquisition, Resources, Supervision, Writing – review & editing. BT: Conceptualization, Funding acquisition, Resources, Supervision, Writing – review & editing. SJ: Conceptualization, Formal analysis, Funding acquisition, Methodology, Resources, Supervision, Validation, Writing – review & editing. DT: Conceptualization, Formal analysis, Funding acquisition, Methodology, Resources, Supervision, Validation, Writing – review & editing.

## Supplementary material

SUPPLEMENTARY FIGURE 1 Goukoviruses distance matrix. Distance matrix of the RdRp gene showing representative viruses of genus *Goukovirus* and samples sequenced in the present study. Light grey to dark shades highlights amino acid distances.

SUPPLEMENTARY FIGURE 2 Peribunyaviruses distance matrix. Distance matrix of the RdRp gene showing representative viruses and samples sequenced in the present study (bold). Light grey to dark shades indicates nucleotide distances.
