# Supplementary figures and images for: Viral diversity and blood-feeding patterns of Afrotropical Culicoides biting midges (Diptera: Ceratopogonidae)
Source: Front Microbiol. 2024 Jan 5;14:1325473. doi: 10.3389/fmicb.2023.1325473 (PMC10797016; doi:10.3389/fmicb.2023.1325473)

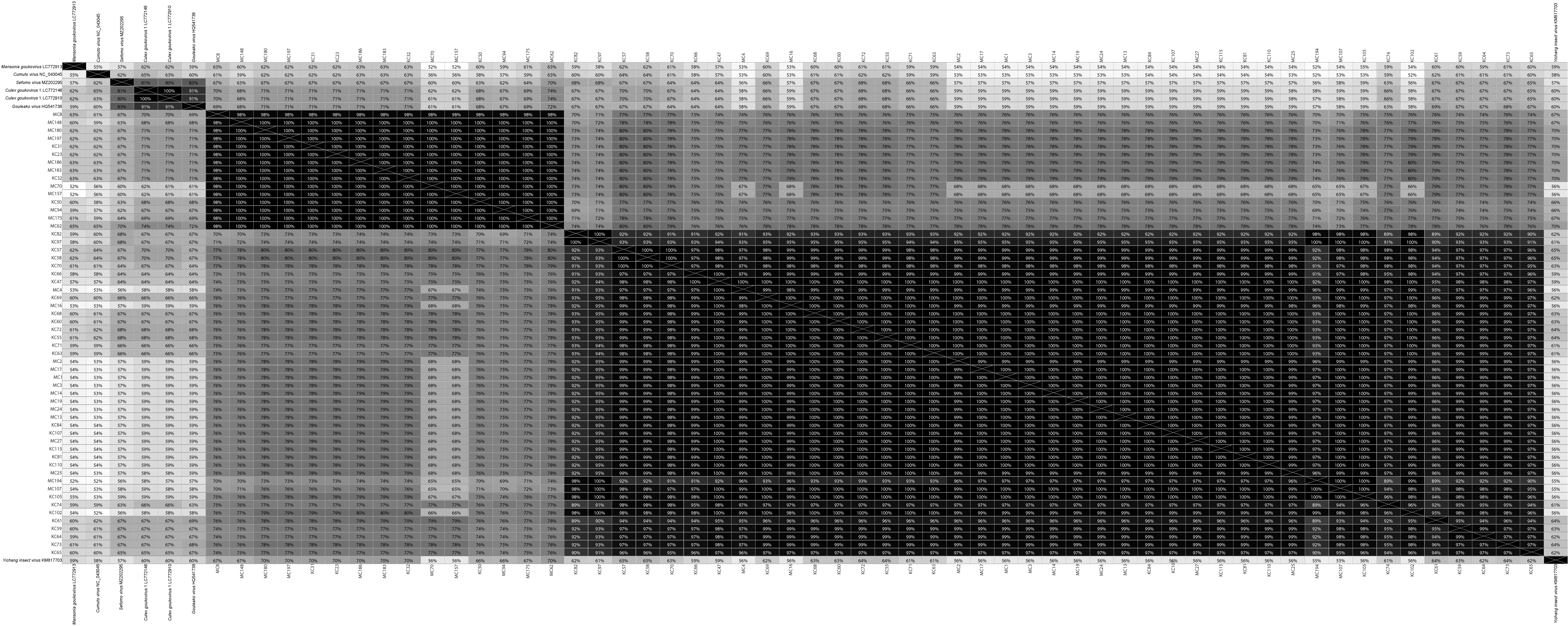

Supplement: SUPPLEMENTARY FIGURE 1 — Goukoviruses distance matrix. Distance matrix of the RdRp gene showing representative viruses of genus Goukovirus and samples sequenced in the present study. Light grey to dark shades highlights amino acid distances. [file Data_Sheet_1.ZIP › Figure S1.tif]

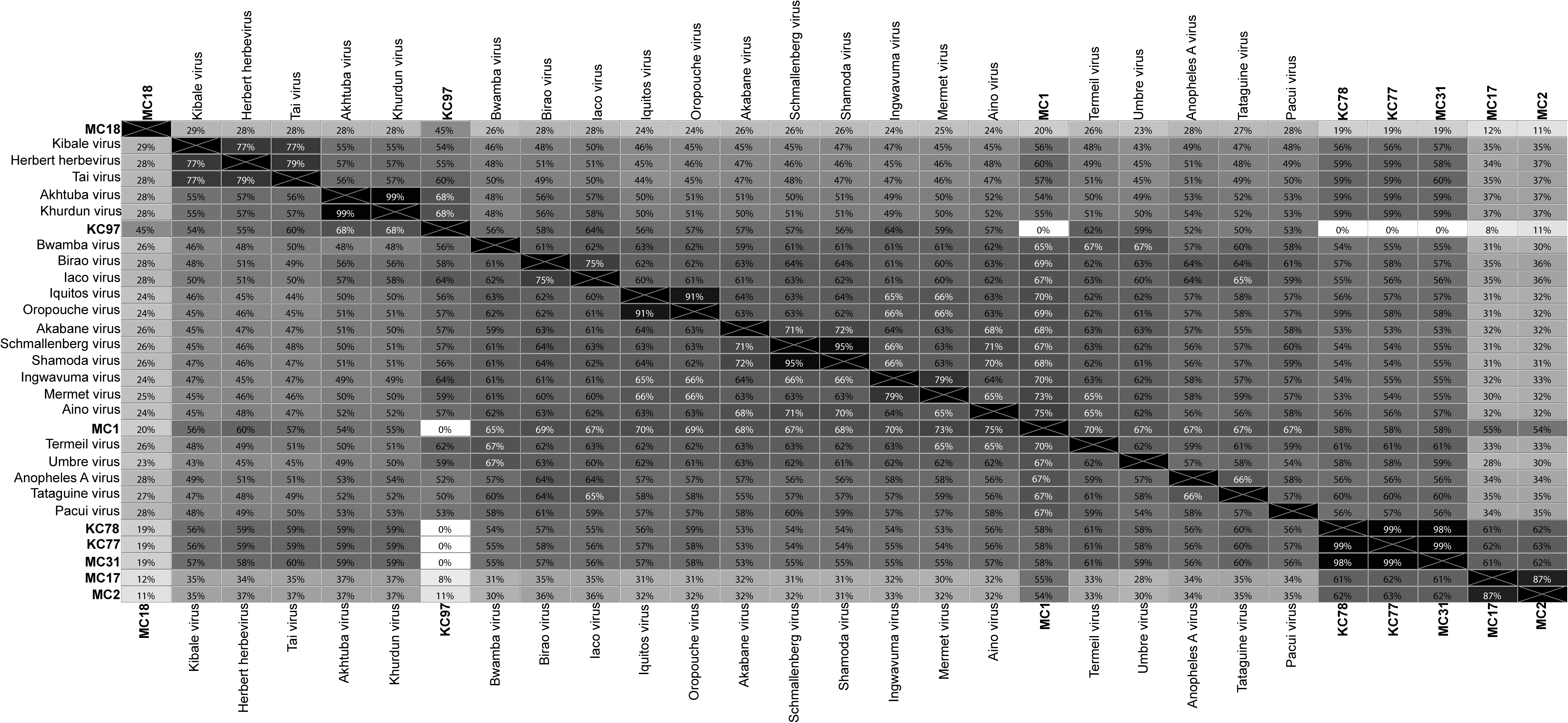

Supplement: SUPPLEMENTARY FIGURE 1 — Goukoviruses distance matrix. Distance matrix of the RdRp gene showing representative viruses of genus Goukovirus and samples sequenced in the present study. Light grey to dark shades highlights amino acid distances. [file Data_Sheet_1.ZIP › Figure S2.tif]
